# Cardiometabolic Biomarkers and Systemic Inflammation in US Adolescents and Young Adults With Latent Tuberculosis Infection: A Population-Based Cohort Study

**DOI:** 10.1093/ofid/ofaf194

**Published:** 2025-03-28

**Authors:** Itai M Magodoro, Ntobeko A B Ntusi, Jennifer Jao, Heather J Zar, Brian L Claggett, Mark J Siedner, Katalin A Wilkinson, Robert J Wilkinson

**Affiliations:** Department of Medicine, University of Cape Town, Observatory, Republic of South Africa; Department of Medicine, University of Cape Town, Observatory, Republic of South Africa; South African Medical Research Council, Tygerberg, Republic of South Africa; ARUA/GUILD Cluster of Research Excellence on Noncommunicable Diseases and Associated Multimorbidity, Cape Town, Republic of South Africa; Division of Pediatric Infectious Diseases, Department of Pediatrics, Feinberg School of Medicine, Northwestern University, Chicago, Illinois, USA; Division of Adult Infectious Diseases, Department of Internal Medicine, Feinberg School of Medicine, Northwestern University, Chicago, Illinois, USA; SAMRC Extramural Unit of Child and Adolescent Health, University of Cape Town, Rondebosch, Republic of South Africa; Harvard Medical School, Boston, Massachusetts, USA; Cardiovascular Division, Brigham and Women's Hospital, Boston, Massachusetts, USA; Harvard Medical School, Boston, Massachusetts, USA; Africa Health Research Institute, Mtubatuba, Republic of South Africa; University of KwaZulu-Natal, Durban, South Africa; Department of Medicine, University of Cape Town, Observatory, Republic of South Africa; Francis Crick Institute, London, UK; Wellcome Discovery Research Platform in Infections, Wellcome CIDRI–Africa and Institute of Infectious Disease and Molecular Medicine, University of Cape Town, Observatory, Republic of South Africa; Department of Medicine, University of Cape Town, Observatory, Republic of South Africa; Francis Crick Institute, London, UK; Wellcome Discovery Research Platform in Infections, Wellcome CIDRI–Africa and Institute of Infectious Disease and Molecular Medicine, University of Cape Town, Observatory, Republic of South Africa; Department of Infectious Diseases, Imperial College, London, UK

**Keywords:** adolescents, cardiometabolic disease, inflammation, LTBI, young adults

## Abstract

**Background:**

*Mycobacterium tuberculosis* (*Mtb*) infection in adults increases incident type 2 diabetes and atherosclerotic cardiovascular disease risk. It is unknown if this cardiometabolic detriment occurs in young people. We investigated whether young persons with latent tuberculosis infection (LTBI) have worse cardiometabolic health than their peers who are uninfected.

**Methods:**

Peripubescent adolescents (12–15 years old) and older adolescents/young adults (16–30 years old) were assessed for LTBI by tuberculin skin testing (induration ≥10 mm). Outcomes included fasting plasma glucose, hemoglobin A_1c_, C-peptide, N-terminal prohormone of brain natriuretic peptide, high-sensitivity cardiac troponin T, C-reactive protein, ferritin, diabetes/prediabetes (fasting plasma glucose ≥5.6 mmol/L and/or hemoglobin A_1c_ ≥5.7%), and homeostatic model of insulin resistance. LTBI cases were propensity score matched 1:4 with controls who were uninfected with tuberculosis (TB) on sociodemographics to estimate adjusted median, mean difference, and odds ratio of cardiometabolic indices.

**Results:**

Seventy-five LTBI cases were matched to 300 peers who were TB uninfected. Among older participants, LTBI was associated with higher inflammation (adjusted median [IQR]: C-reactive protein, 0.22 mg/dL [0.05–0.34] vs 0.11 [0.04–0.35], *P* = .027; ferritin, 55.0 ng/mL [25.1–90.3] vs 41.1 [29.5–136.2], *P* = .047) but not among peripubescent adolescents. No meaningful differences were observed in fasting plasma glucose (adjusted mean difference [95% CI], −0.05 mmol/L [−.22 to .12]; *P* = .57), hemoglobin A_1c_ (0.0% [−.17% to .17%], *P* = .98), diabetes/prediabetes prevalence (adjusted odds ratio [95% CI], 0.9 [.29–2.29]; *P* = .85), insulin secretion/resistance, N-terminal prohormone of brain natriuretic peptide, or high-sensitivity cardiac troponin T by LTBI status.

**Conclusions:**

Older adolescents and young adults with LTBI had higher inflammation than those without LTBI, while cardiometabolic profiles were similar. Unlike that in adults, *Mtb* infection in young people may not be associated with cardiometabolic derangement, though the long-term consequences of chronic inflammation require further study.

*Mycobacterium tuberculosis* (*Mtb*) infection, active and latent, has been linked with cardiometabolic disorders in adults [1–5]. A tuberculosis (TB) diagnosis in adults confers 2-fold increased risk (relative risk, 1.9; 95% CI, 1.4–2.7) for future cardiovascular morbidity and mortality according to a 2020 meta-analysis [1], while prospective observational studies find up to 5-fold increased risk for incident diabetes [2, 4]. Human and murine TB is associated with hypercoagulability [6], elevated blood pressure [7], endothelial and microvascular dysfunction, cardiac hypertrophy and fibrosis [8], and increased insulin resistance [3], among others, driven by an amplified and/or dysregulated inflammatory response to *Mtb* antigens [9]. Together, these findings suggest that TB may be a novel risk factor for type 2 diabetes mellitus (diabetes) and atherosclerotic cardiovascular disease (CVD). TB may itself increase cardiometabolic risk because of its long latency and often delayed clinical diagnosis, resulting in chronic inflammation. Indeed, Critchley et al [10] report higher CVD incidence in adults in the United Kingdom and United States with TB as compared with peers without, 2 years before and 2 years after TB diagnosis. Incident CVD risk was markedly increased in the acute period—specifically, 3 months before and after TB diagnosis (adjusted incident rate ratio [95% CI]: United States, 3.2 [2.2–4.4]; United Kingdom, 1.6 [1.2–2.1]). However, it is unknown if this cardiometabolic detriment also occurs in the young.

Globally, an estimated 67 million adolescents (<15 years old) have latent TB infection (LTBI) [11], and 1.8 million adolescents and young adults (10–24 years old) develop active TB annually, representing 17% of all new TB disease diagnoses [12, 13]. If *Mtb* infection is cardiometabolically detrimental, this young population may be vulnerable to chronic disease and poor health in adulthood. *Mtb* exposure in early life will coincide with an accumulating burden of traditional risk factors: smoking, inadequate physical activity, excess calorie intake, and air pollution, among others [14]. Although overt cardiometabolic disease is considered to affect only adults, it does have its beginnings in the first decade of life, where its antecedents depend on the number, duration, and intensity of risk factors [15]. Yet, the inflammatory response to *Mtb* infection varies with age. Peripubescent children and adolescents exhibit a balance of protective and tissue-damaging immunity, unlike younger children (<5 years) or older people (>15 years) [16]. This period, dubbed the “wonder years” of TB protective immunity, has the lowest risk of *Mtb* infection progressing to active TB across the life span [17, 18]. Active TB in this age group typically presents as paucibacillary intrathoracic disease, primarily involving mediastinal lymph nodes rather than lung parenchyma [18–20]. This is relatively benign as compared with disseminated disease in young children or destructive pulmonary disease in older adolescents and adults [18–20]. However, the cardiometabolic correlates of this age-varying immunity to TB are unknown.

Therefore, understanding the nexus of *Mtb* infection, cardiometabolic health, and age in young persons could be key to preventing TB-related diabetes and/or cardiovascular complications in adulthood, ultimately reducing their future burden. Conversely, if this association is absent, identifying relevant protective factors is crucial. Our study's objective, therefore, was to evaluate the hitherto underexamined cardiometabolic health of young people (12–30 years) with LTBI and its age-related variations as compared with matched peers uninfected with *Mtb*.

## METHODS

We followed the STROBE guidelines (Strengthening the Reporting of Observational Studies in Epidemiology) [21] in the conduct and reporting of our analyses.

### Data Sources

This study used data from the National Health and Nutrition Examination Survey (NHANES), a series of biennial cross-sectional health examinations of US children and adults. NHANES participants complete standardized questionnaires, physical examination, and blood testing. Study protocols, including ethics and informed consent, are described elsewhere [22]. As NHANES data are anonymized and publicly available, institutional review board approval was not sought for this study.

### Analytic Sample Selection

We analyzed NHANES participants aged 12 to 30 years, excluding those with missing data on tuberculin skin testing (TST), age, sex, race and ethnicity, household food security, physical activity, serum cotinine, and body mass index (Supplementary Figure 1)—covariates used for propensity score (PS) matching. Other exclusions were those using insulin. Because there were no data to distinguish types 1 and 2 diabetes, excluding current insulin users in this young population likely removed any type 1 diabetes cases. We also excluded those with evidence of bacillus Calmette-Guérin (BCG) vaccination scars. Prior BCG vaccination is an important cause of false-positive TST reactions [23]. BCG vaccination status was reported in the 1999–2000 NHANES cycle but not the 2011–2012 NHANES cycle. The 1999–2000 cycle was used for primary analysis and the 2011–2012 cycle for secondary analysis.

### Study Measures

#### Latent TB Infection

TST was undertaken with a tuberculin purified protein derivative (Tubersol; Sanofi). Induration ≥10 mm indicated LTBI, as measured 48 to 72 hours after intradermal placement of the purified protein derivative on the volar forearm surface [24]. Participants were also asked if they had ever had a TB skin test (tine or tuberculin); if positive, were they ever prescribed TB preventive therapy; had they ever had active TB; if history of active TB, were they ever prescribed anti-TB drug treatment; and had they ever lived in a household with an active TB contact. The presence of any one of these was considered likely prior TB vs, for example, nontuberculous mycobacteria exposure. Nontuberculous mycobacteria, with BCG vaccination, is another important cause of false-positive TST reactivity [23]. Chest radiographs and current TB symptoms were not collected. No participants were HIV seropositive.

#### Cardiometabolic Indices and Inflammation Markers

Fasting plasma glucose (FPG), fasting serum insulin, and C-peptide concentrations were measured after an overnight fast ≥9 hours. Glycated albumin (Alb_1c_) and hemoglobin (HbA_1c_), as well as lipid subfractions (high- and low-density lipoprotein and triglycerides), were measured in nonfasting blood. N-terminal prohormone of brain natriuretic peptide (limit of detection, ≥5 pg/mL; Roche Diagnostics), high-sensitivity cardiac troponin T (limit of detection, ≥3 ng/L; Roche Diagnostics), cystatin C (limit of detection, ≥0.23 mg/dL; Dade Behring), high-sensitivity C-reactive protein (hsCRP; Dade Behring), and ferritin (BioRad Laboratories) were measured in serum [25]. Neutrophil-lymphocyte ratio was calculated from a complete blood count (Beckman Coulter). Seated systolic and diastolic blood pressure was measured 4 times with a mercury sphygmomanometer and the last 3 readings averaged.

Homoeostasis model assessment 2 of insulin resistance (HOMA2-IR) was calculated by FPG and fasting C-peptide (or fasting insulin for the 2011–2012 NHANES) with the homoeostasis model assessment calculator (University of Oxford) [26]. Diabetes was defined as FPG ≥7.0 mmol/L or HbA_1c_ ≥6.5% and prediabetes as FPG of 5.6 to 6.9 mmol/L or HbA_1c_ of 5.7% to 6.4% [27]. Due to the low number of diabetes cases, diabetes and prediabetes were combined as dysglycemia (diabetes/prediabetes).

#### Demographic, Behavioral, and Biophysical Measures

Participants were categorized as peripubescent adolescents (12–15 years) or older adolescents and young adults (ie, older participants; 16–30 years). Sex was self-reported as male or female. Race was categorized as Hispanic or non-Hispanic, with the latter merging non-Hispanic White, Black, and Asian/other. Country of birth was classified as either US or non-US. Socioeconomic status was assessed by household food security, grouped as food secure or insecure. Food-insecure households reported relying on low-cost food, running out of food, or skipping and/or reducing the size of meals in the preceding 12 months. Sedentary time (hours/day) was estimated from reported time spent watching television or movies or using computers or games the previous day. Serum cotinine, a nicotine biomarker, assessed tobacco smoke exposure from primary and secondhand smoking [28]. Weight and height were measured to calculate body mass index.

### Statistical Analysis

#### Matching and Covariate Selection

To improve comparability and reduce bias, participants with LTBI were PS matched 1:4 to peers uninfected with TB by nearest neighbor matching within a 0.1 caliper and without replacement.

PS was estimated via multivariable logistic regression with LTBI status as the outcome and with the predictors being age, age group (12–15 and 16–30 years), sex, race, country of birth, household food security (representing socioeconomic status), sedentary time, serum cotinine (representing smoking), and body mass index. These were selected as potential confounders of the LTBI-metabolism relationship and/or metabolic risk factors. Postmatch balance was assessed by standardized mean difference and the Kolmogorov-Smirnov statistic for each baseline covariate. A standardized mean difference <0.10 and Kolmogorov-Smirnov <0.05 denoted inconsequential residual bias (Supplementary Figure 2).

Age group stratification was based on significant linear effects of LTBI status (*Mtb* uninfected vs LTBI) on hsCRP and ferritin, incorporating interaction terms between TB status and age group (Supplementary Table 1). Our a priori hypothesis was that *Mtb*-related inflammation, and thus cardiometabolic risk, varies with age. Primary outcomes were FPG, Alb_1c_, HbA_1c_, HOMA2-IR, and dysglycemia prevalence as indices of glucose metabolism. However, no significant *Mtb* status × age group interactions were found for these (interaction term, *P* ≥ .19), so only main effects of LTBI are reported. For the secondary outcomes, main effects of LTBI and age group are reported.

Generalized linear models assessed differences (95% CI) by LTBI status in primary outcomes via identity and logit link functions for continuous and dichotomous outcomes, respectively. Analyses used unmatched samples without confounder adjustment and PS-matched samples. Median differences in N-terminal prohormone of brain natriuretic peptide, high-sensitivity cardiac troponin T, cystatin C, CRP, low-density lipoprotein, triglycerides, and systolic blood pressure by LTBI status and age group were examined as secondary outcomes by quantile regression in unmatched and PS-matched samples. Distributions were visually compared by box-and-whisker plots. Descriptive statistics are presented by LTBI status and variable scale: mean (SD), median (IQR), or number (percentage).

#### Secondary Analysis

We tested the consistency of our results in a different cohort collected a decade later (ie, 2011–2012 NHANES sample) focusing on glucose metabolism. We replicated the PS-matching procedure using similarly defined covariates as in the primary analysis and repeated the PS-matched generalized linear model analyses (Supplementary Table 2). Of note, BCG vaccine status was not reported in the 2011–2012 NHANES. Participants in the 2011–2012 NHANES cycle also completed an oral glucose tolerance test from which we obtained 2-hour postload plasma glucose in addition to FPG, fasting insulin, diabetes/prediabetes (FPG ≥5.6 mmol/L or HbA_1c_ ≥5.7% and/or postload plasma glucose ≥7.8 mmol/L), and HOMA2-IR.

Analyses were conducted in R version 3.6.3 (R Foundation for Statistical Computing) and Stata version 17.0 (StataCorp), with Bonferroni-corrected *P* values <.007 considered statistically significant for the primary end points. Matching was performed with the MatchIt package [29] and covariate balance assessed with the cobalt package [30], both in R.

## RESULTS

### Baseline Characteristics of Matched Sample

The analytic sample consisted of 2342 participants (Supplementary Figure 1). PS matching achieved a balance of baseline covariates (Table 1, Supplementary Figure 2) and yielded an effective sample size of 375, of which 75 participants had LTBI and 300 were controls uninfected with *Mtb*. The mean (SD) age of participants in the matched sample was 18.3 (5.5) years for those with LTBI and 18.0 (5.5) years for those uninfected with *Mtb*. The majority of participants were male (62.1%), Hispanic (76.3%), and born outside the United States (65.9%). Participants were of comparatively low socioeconomic status given the common frequency of household food insecurity (33.1%). Although comparatively similar proportions of those with LTBI (72.6%) and their peers uninfected with TB (66.0%, *P* = .33) had a prior TB skin test, the former more frequently reported a positive test result (28.0% vs 4.3%, *P* < .001; Table 2). Overall, participants with LTBI (33.3%) were about 5 times as likely to have an indicator of likely prior TB exposure than controls uninfected with TB (7.3%, *P* < .001).

**Table 1.** Sociodemographic and Behavioral Characteristics of Adolescent and Young Adult Participants (12–30 Years Old) According to LTBI Status Before and After PS Matching: US National Health and Nutrition Examination Survey, 1999–2000

	Unmatched			PS Matched		
Characteristic	*Mtb* Uninfected	LTBI	SMD	*Mtb* Uninfected	LTBI	SMD
No.	2142	75		300	75	
Age, y, mean (SD)	17.8 (4.9)	18.3 (5.5)	0.087	18.0 (5.5)	18.3 (5.5)	0.050
12–15	961 (38.5)	32 (42.7)	0.085	136 (45.3)	32 (42.7)	…
16–30	1534 (61.5)	43 (57.3)	…	164 (54.7)	43 (57.3)	0.054
Female sex	1292 (51.8)	29 (38.7)	0.27	113 (37.7)	29 (38.7)	0.021
Race/ethnicity						
Hispanic	1100 (44.1)	56 (74.3)	0.66	230 (76.7)	56 (74.7)	0.070
Non-Hispanic	1395 (55.9)	19 (25.3)	…	70 (23.3)	19 (25.3)	0.047
Country of birth						
US	442 (17.7)	25 (33.3)	1.14	103 (34.3)	25 (33.3)	0.023
Non-US	2053 (82.3)	50 (66.7)	…	197 (65.7)	50 (66.7)	…
Household food security						
Secure	1958 (78.5)	50 (66.7)	0.27	201 (67.0)	50 (66.7)	0.007
Insecure	537 (21.5)	25 (33.3)	…	99 (33.0)	25 (33.3)	…
Sedentary time, h/d, mean (SD)	3.4 (1.6)	3.5 (1.5)	0.020	3.5 (1.6)	3.5 (1.5)	0.004
Cotinine, ng/mL, median (IQR)	0.16 (0.04–1.98)	0.05 (0.04–1.13)	0.27	0.08 (0.04–1.06)	0.05 (0.04–1.59)	0.075
BMI, kg/m^2^, mean (SD)	25.0 (6.3)	24.6 (5.9)	0.095	24.7 (5.6)	24.6 (5.9)	0.016

Values are presented as No. (%) unless indicated otherwise.

Abbreviations: BMI, body mass index; LTBI, latent tuberculosis infection; *Mtb*, *Mycobacterium tuberculosis*; PS, propensity score; SMD, standardized mean difference.

**Table 2.** TB-Related History of Unmatched and PS-Matched Adolescent and Young Adult Participants (12–30 Years Old): US National Health and Nutrition Examination Survey, 1999–2000

	Unmatched			PS Matched		
Characteristic	*Mtb* Uninfected	LTBI	*P* Value	*Mtb* Uninfected	LTBI	*P* Value
No.	2142	75		300	75	
Tuberculin skin induration, mm						
Mean (SD)	0.44 (1.25)	13.9 (3.23)	<.001	0.85 (1.89)	13.9 (3.23)	<.001
Median (IQR)	0.4 (0.0–1.1)	13.5 (12.0–15.7)	<.001	0.0 (0–0)	13.5 (12.0–15.7)	<.001
Ever had TB skin test	1723 (71.5)	53 (72.6)	.94	192 (66.0)	53 (72.6)	.33
Had negative result	1665 (66.7)	30 (40.0)	<.001	178 (59.3)	30 (40.0)	<.001
Had positive result	50 (2.0)	21 (28.0)	<.001	13 (4.3)	21 (28.0)	<.001
Unknown/missing result	780 (31.3)	24 (32.0)	…	109 (36.3)	24 (32.0)	…
Prescribed TBT	29 (1.2)	17 (22.7)	<.001	9 (3.0)	17 (22.7)	<.001
Ever had active TB	5 (0.2)	2 (2.7)	.004	2 (0.7)	2 (2.7)	.041
Prescribed ATT	2 (0.1)	2 (2.7)	<.001	2 (0.7)	2 (2.7)	.23
Ever had household active TB contact	44 (1.8)	8 (10.8)	<.001	10 (3.4)	8 (10.8)	.001
Any indicator of likely prior TB exposure^a^	93 (3.7)	25 (33.3)	<.001	22 (7.3)	25 (33.3)	<.001

Values are presented as No. (%) unless indicated otherwise.

Abbreviations: ATT, antituberculosis therapy; LTBI, latent tuberculosis infection; *Mtb*, *Mycobacterium tuberculosis*; PS, propensity score; SMD, standardized mean difference; TB, tuberculosis; TBT, tuberculosis preventive therapy.

^a^Presence of any 1 of the following: positive TB skin test result, active TB, prescribed ATT for active TB, and household active TB contact.

### Distribution of Cardiometabolic Biomarkers

**Figure 1.**
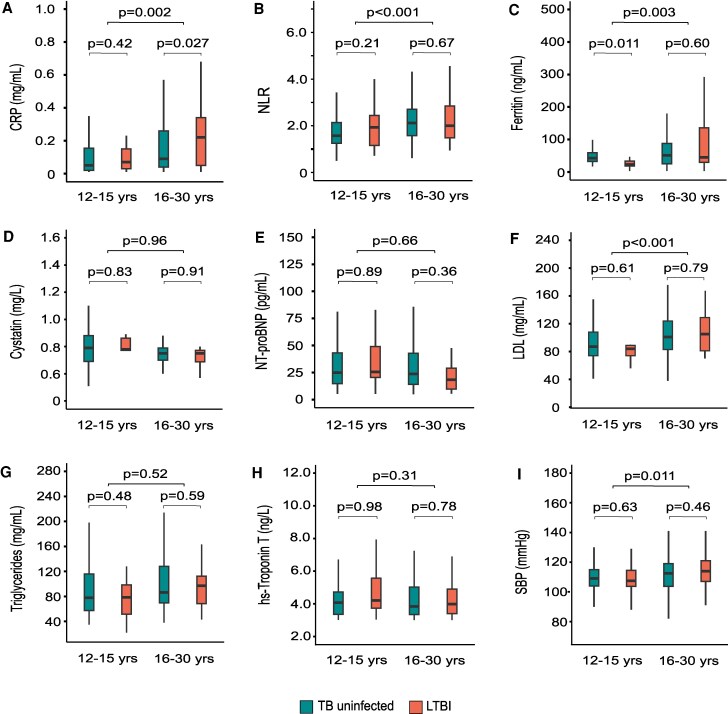
Cardiometabolic profiles of propensity score–matched adolescent and young adult participants (12–30 years) according to latent tuberculosis infection status and stratified by age, US National Health and Nutrition Examination Survey, 1999–2000. *P* values are for differences in median values according to quantile regression models. Matching is based on age, sex, race, country of birth, household food, sedentary time, serum cotinine, and body mass index. Data are presented as median (line), IQR (box), and 95% CI (whiskers). Abbreviations: BNP, brain natriuretic peptide; CRP, C-reactive protein; HDL, high-density lipoprotein cholesterol; hs-Troponin T, high-sensitivity cardiac troponin T; NLR, neutrophil-lymphocyte ratio; NT-proBNP, N-terminal prohormone of brain natriuretic peptide; SBP, systolic blood pressure.

*Mtb* status × age group interaction effects postmatch were statistically significant for hsCRP (β-coefficient [95% CI], 0.12 [.01–.25]; *P* = .031) and ferritin (0.49 [.03–.95], *P* = .038), not neutrophil-lymphocyte ratio (−0.12 [−.35 to .10], *P* = .29; Supplementary Table 1). As such, LTBI was associated with higher hsCRP (median [IQR], 0.22 mg/dL [0.05–0.34] vs 0.11 [0.04–0.35]; *P* = .027] and ferritin (55.0 ng/mL [25.1–90.3] vs 41.1 [29.5–136.2], *P* = .047) as compared with controls uninfected with TB, though among only older participants. In peripubescent adolescents, those with LTBI had lower ferritin (23.0 ng/mL [18.5–33.5]) than controls (32.7 ng/mL [21.5–48.2], *P* = .011) but comparable hsCRP (0.08 mg/dL [0.04–0.36] vs 0.05 [0.02–0.17], *P* = .42; Figure 1). With the exception of higher low-density lipoprotein (101 mg/mL [81–124] vs 86 [74–108], *P* < .001) and systolic blood pressure (113 mm Hg [104–120] vs 109 [104–115], *P* = .011) in older participants than peripubescent adolescents, there were neither statistically nor clinically significant differences by *Mtb* status or age group in myocardial stress (N-terminal prohormone of brain natriuretic peptide) or necrosis (high-sensitivity cardiac troponin T), cardiovascular risk (cystatin C, systolic blood pressure), or triglycerides (Figure 1).

### Glucose Metabolism Outcomes

The odds of having dysglycemia (diabetes/prediabetes) were similar with or without LTBI before PS matching (odds ratio [OR], 1.29 [95% CI, .45–2.95]; *P* = .59) and after (adjusted OR, 0.9 [.29–2.29]; *P* = .85; Table 3).

**Table 3.** Associations Between LTBI and Glucose Metabolism Indices Among PS-Matched Adolescent and Young Adult Participants (12–30 Years Old): Unweighted US National Health and Nutrition Examination Survey, 1999–2000

	Mean (95% CI)^a^			
Outcome	*Mtb* Uninfected	LTBI	Effect Size (95% CI)^b^	*P* Value^c^
Fasting glucose, mmol/L				
Unmatched	5.0 (4.9–5.1)	5.1 (4.8–5.4)	0.04 (−.25, .33)	.79
PS matched	5.1 (4.9–5.4)	5.1 (4.8–5.4)	0.05 (−.22, .12)	.57
HbA_1c_, %				
Unmatched	5.1 (5.0–5.1)	5.1 (4.9–5.2)	0.0 (−.10, .10)	.93
PS matched	5.0 (5.0–5.1)	5.1 (4.9–5.2)	0.0 (−.17, .17)	.98
Glycated albumin, %				
Unmatched	13.8 (13.0–14.5)	12.7 (8.5–17.0)	−1.1 (−5.4, 3.3)	.65
PS matched	14.2 (12.2–16.3)	12.7 (8.5–17.0)	−1.5 (−5.6, 3.0)	.52
Diabetes/prediabetes prevalence,^d^ %				
Unmatched	5.3 (4.4–6.2)	6.7 (2.8–15.0)	1.29 (.45, 2.95)	.59
PS matched	7.3 (4.9–10.9)	6.7 (2.8–15.0)	0.9 (.29, 2.29)	.85
Fasting insulin, pmol/L				
Unmatched	82.3 (78.6–86.1)	79.2 (57.6–100.8)	−3.1 (−25.0, 18.8)	.78
PS matched	86.5 (78.2–94.7)	79.2 (57.6–100.8)	−7.2 (−26.5, 12.0)	.46
Fasting C-peptide, nmol/L				
Unmatched	0.73 (.71–.75)	0.73 (.61–.85)	0.0 (−.12, .12)	.98
PS matched	0.78 (.73–.84)	0.73 (.61–.85)	−0.05 (−.18, .09)	.51
HOMA2-IR				
Unmatched	1.57 (1.53–1.61)	1.60 (1.36–1.85)	0.03 (−.21, .28)	.79
PS matched	1.72 (1.60–1.83)	1.60 (1.37–1.84)	−0.11 (−.38, .15)	.41

Models are PS matched on age, sex, race, country of birth, household food, sedentary time, serum cotinine, and body mass index.

Abbreviations: HbA_1c_, hemoglobin A_1c_; HOMA2-IR, homoeostasis model assessment 2 of insulin resistance; LTBI, latent tuberculosis infection; *Mtb*, *Mycobacterium tuberculosis*; PS, propensity score.

^a^Mean (95% CI) values are postestimation margins from generalized linear models with identity function for continuous outcomes and logit function for dichotomous outcome (diabetes/prediabetes).

^b^Effect estimates are mean differences for continuous outcomes or odds ratio for dichotomous outcome (diabetes/prediabetes) with the *Mtb***-**uninfected group as reference.

^c^Statistically significant at Bonferroni-corrected *P* < .007.

^d^Diabetes/prediabetes defined as fasting plasma glucose ≥5.6 mmol/L or HbA_1c_ ≥5.7%.

Insulin secretion as measured by fasting C-peptide was similar between participants with LTBI and controls (adjusted mean difference [95% CI], −0.05 nmol/L [−.18 to .09]; *P* = .51), as was insulin resistance measured by HOMA2-IR (−0.11 [−.38 to .15], *P* = .41). We also did not find any statistically or clinically significant differences in glycemia—immediate (FPG, −0.05 mmol/L [−.22 to .12]; *P* = .57), medium range (Alb_1c_, −1.5% [−.56% to 3.0%]; *P* = .52), or long range (HbA_1c_, 0.0% [−.17% to .17%]; *P* = .98)—following PS matching. Comparisons in the unmatched sample found similar results.

### Secondary Analysis

When PS matching was repeated with the 2011–2012 NHANES cycle, we achieved a matched secondary sample of 345. Of these, 69 had LTBI and 276 were *Mtb* uninfected (Supplementary Table 2). The secondary analytic sample was slightly older (mean age, 21.6 vs 18.2 years) and had a higher proportion of non–US-born participants (75.1% vs 65.9%) than the primary sample (1999–2000 NHANES; Table 1). Notwithstanding, the odds of diabetes/prediabetes in the secondary sample were similar (adjusted OR [95% CI], 1.19 [.62–2.19]; *P* = .59) for the 2 exposure groups (Table 4). Neither were there notable differences in FPG (adjusted mean difference [95% CI], −0.07 mmol/L [−.48 to .34]; *P* = .75), postload plasma glucose (−0.36 mmol/L [−1.15 to .44], *P* = .38), HbA_1c_ (−0.04% [−.22% to .12%], *P* = .57), or HOMA2-IR (−0.88 [−.55 to .38], *P* = .73).

**Table 4.** Secondary Analysis: PS-Matched Associations Between LTBI and Glucose Metabolism Indices Among Adolescent and Young Adult Participants (12–30 Years Old): US National Health and Nutrition Examination Survey, 2011–2012

	Mean (95% CI)^a^			
Outcome	*Mtb* Uninfected	LTBI	Effect Size (95% CI)^b^	*P* Value
Fasting glucose, mmol/L	5.4 (5.2–5.5)	5.3 (4.9–5.7)	−0.07 (−.48, .34)	.75
Postload glucose,^c^ mmol/L	5.8 (5.4–6.2)	5.5 (4.7–6.2)	−0.36 (−1.15, .44)	.38
HbA_1c_, %	5.3 (5.2–5.4)	5.3 (5.1–5.4)	−0.04 (−.22, .12)	.57
Fasting insulin, pmol/L	85.0 (72.1–98.0)	101.1 (74.9–128.0)	16.2 (−12.9, 45.4)	.28
Diabetes/prediabetes prevalence,^d^ %	20.3 (16.9–25.4)	23.2 (14.7–34.5)	1.19 (.62, 2.19)	.59
HOMA2-IR	1.65 (1.44–1.86)	1.57 (1.14–1.99)	−0.88 (−.55, .38)	.73

Models are PS matched 1:4 on age, sex, race, country of birth, household food, sedentary time, serum cotinine, and body mass index.

Abbreviations: HbA_1c_, hemoglobin A_1c_; HOMA2-IR, homoeostasis model assessment 2 of insulin resistance; LTBI, latent tuberculosis infection; *Mtb*, *Mycobacterium tuberculosis*; PS, propensity score.

^a^Mean (95% CI) values are postestimation margins from generalized linear models with identity function for continuous outcomes and logit function for dichotomous outcome (diabetes/prediabetes) in the PS-matched sample without covariate adjustment.

^b^Effect estimates are mean differences for continuous outcomes or odds ratio for dichotomous outcome (diabetes/prediabetes) with the *Mtb***-**uninfected group as reference.

^c^2-hour plasma glucose following standard 75-g glucose oral tolerance rest.

^d^Diabetes/prediabetes defined as fasting plasma glucose ≥5.6 mmol/L, HbA_1c_ ≥5.7%, and/or postload glucose ≥7.8 mmol/L.

## DISCUSSION

We compared cross-sectional cardiometabolic biomarkers among young persons (12–30 years) in the United States with LTBI vs their peers uninfected with *Mtb*. We hypothesized that *Mtb* infection, by driving inflammation, is associated with worse profiles of glucose metabolism and cardiovascular status in an age-dependent manner. Unlike older participants (16–30 years)—among whom LTBI was associated with higher inflammation of, as yet, undetermined clinical significance—peripubescent adolescents (12–15 years) had no evidence of LTBI-related differences in inflammation. In fact, the acute-phase reactant, ferritin, was reduced. We had insufficient data to assess diabetes/prediabetes, a relatively rare outcome in such young people. However, we did not find any clinically meaningful differences in the remainder of glucose metabolism readouts in relation to LTBI. These findings were replicated in the secondary analysis from a separate survey, underscoring their robustness. The biomarkers assessed in our study represent wide domains of cardiometabolic health, such as myocardial stress and necrosis, cardiorenal status, lipid metabolism, blood pressure, glycemia (short, medium, and long range), glucose load handling, and insulin sensitivity. That latent *Mtb* infection in young persons may not be associated with cardiometabolic derangement requires confirmation in larger samples and especially in settings with high TB burden. Whether this is also the case for active TB remains to be established.

*Mtb* infection in adults associates with transient impairment of glucose regulation and new-onset diabetes [2, 4]. Transient hyperglycemia occurs during active TB and resolves with antitubercular drug treatment. It is debated whether this is just stress hyperglycemia [31], as occurs in other severe systemic infections, or it reflects true metabolic dysfunction, especially when it outlasts antitubercular drug treatment. Hyperglycemia that is not in the diabetic range and persists up to 4 months post-TB has been reported in patients treated for pulmonary TB [32]. Notably, these patients did not have diabetes at the time of TB diagnosis or before. Regarding overt diabetes developing in the post-TB period, it is also not clear whether this is presaged by any dysglycemia, transient or otherwise, during active TB.

A study of a UK nationwide primary care adult cohort reported adjusted incidence rate ratios for new-onset diabetes of 5.7 following pulmonary TB and 4.7 following extrapulmonary TB as compared with cohorts unexposed to TB [4]. Average post-TB follow-up was 4 years. A US-wide study found higher diabetes incidence in adults with reactive TST and/or interferon-γ release assay than in nonreactive peers (adjusted HR, 1.2; 95% CI, 1.2–1.3) [2]. We are not aware of comparable studies in children and adolescents. Salindri et al examined the Taiwan national health insurance database (2002–2013), which covers 99.9% of the national population, and found a diabetes incidence rate of 0.25 (95% CI, .15–.43) per 1000 person-years in persons aged 15 to 24 years previously treated for TB [33]. Their study did not include a control group uninfected with *Mtb*. However, diabetes incidence in a similar age group (10–24 years) in the general population in Taiwan was separately estimated to be 0.12 (95% CI, .11–.12) per 1000 person-years with the same national health insurance database (2000–2009) [34].

*Mtb* infection results in the activation of proinflammatory cytokines, free oxygen radicals, and advanced glycation end products, leading to matrix destruction, cell death, fibrosis, and ultimately disordered tissue remodeling. In the cardiovascular system, these drive insulin resistance, hypercoagulability, elevated blood pressure, and endothelial and microvascular dysfunction. These intermediate states are thought to mediate the link between TB and atherosclerotic CVD in adults [9, 35]. Cross-sectional studies in Saudi Arabia [36], Egypt [37], Uganda [38], Peru [38], and the United States [39] note increased odds of prevalent atherosclerotic CVD with LTBI. A 2023 Canadian prospective cohort study (1985–2019) including nearly 50 000 adults followed for a median 19 years reported higher risk of incident atherosclerotic CVD with LTBI as compared with no TB (adjusted HR, 1.08; 95% CI, .99–1.18) [40]. We did not, however, find any evidence that young persons with LTBI had worse profiles of myocardial stress (N-terminal prohormone of brain natriuretic peptide) or necrosis (high-sensitivity cardiac troponin T), cardiorenal function (cystatin C), or blood pressure, for example, than the controls uninfected with *Mtb*. Only systemic inflammation (hsCRP) was significantly higher among the older participants with LTBI (16–30 years). It is possible to hypothesize that chronically elevated hsCRP in those with LTBI may predispose them later in adulthood toward developing CVD.

Although our findings require replication, we speculate about possible explanations. Puberty varies in age of onset, and the 12- to 15-year age band in our cohort may have spanned the peripubescent “wonder years” of protective immunity against TB [16]. Because this age group effectively contains *Mtb* within granuloma, LTBI in these young persons is likely accompanied by optimal balance between pro- and anti-inflammatory responses [41]. The reduced ferritin among peripubescent adolescents with LTBI in our study may reflect this. We thus postulate that this balanced immune state manifests as the lack of cardiometabolic derangement among our peripubescent adolescents. Yet, *Mtb* infection in older adolescents and adults is accompanied by amplified and/or dysregulated inflammatory responses to mycobacterial antigens with tissue destruction [16, 17]. Indeed, we found higher inflammation with LTBI among our older participants (16–39 years). This, however, was not accompanied by evidence of cardiometabolic detriment in this age group.

Future confirmatory work with larger samples including latent and past active TB is required. In TB-endemic areas such as sub-Saharan Africa and Southeast Asia, pediatric and adolescent TB overlaps with malnutrition and/or HIV. How and if these TB comorbidities affect cardiometabolism also requires urgent attention. Coincidentally, these regions are emerging epicenters of premature-onset adult diabetes and CVDs [42]. A wider array of immune biomarkers will help in elucidating potential protective mechanisms [43]. Imaging modalities and physiologic assessments, such as cardiac magnetic resonance and pulse tonometry, are sensitive measures of preclinical damage. Approaches combining these with immune -*omics* would shed light into phenotypes and mechanisms of TB-related cardiometabolism in adolescents.

### Strengths and Limitations

Our study is among the first to explore the potential cardiometabolic detriment of LTBI in young persons. Participants of Hispanic race/ethnicity and non-US birth made up the majority of our sample, reflecting TB epidemiology in the United States and having implications for the generalizability of our findings. Our sample size of persons with LTBI was small for a comparatively rare outcome such as type 2 diabetes in a young population. As such, we obtained imprecise confidence intervals (adjusted OR, 0.94; 95% CI, .29–2.29) for prevalent dysglycemia with LTBI vs controls uninfected with *Mtb*. Because overt type 2 diabetes is rare in this age cohort, we hypothesized that any *Mtb*-related deleterious cardiometabolic effects might instead manifest as intermediate substrates, such as worse FPG, HbA_1c_ and Alb_1c_, and/or as suboptimal insulin sensitivity and glucose load handling, among others. Whereas FPG is immediate, Alb_1c_ (2–3 weeks) [44] and HbA_1c_ (2–3 months) are markers of medium- and long-range glycemic control. We did not find any clinically meaningful differences in these indices either. Insulin secretion and resistance were also similar, as was glucose tolerance. Our study may very well have been underpowered to detect these differences, including *Mtb* status × age group interaction effects. That these findings were largely replicated in secondary analyses, however, mitigates against this. Another limitation was the use of surrogate measures of cardiometabolic health. Imaging and physiologic assessments would have been more specific and sensitive markers of preclinical CVD. Data on TB-related symptoms, chest radiographs, and sputum examinations in conjunction with TST would have enabled better stratification into those who eliminated their TB infection, controlled their TB infection, or had subclinical TB infection. Excluding participants with BCG scars ameliorated potential misclassification biases from false-positive TST results. Finally, TB and diabetes have a bidirectional relationship, and as a cross-sectional study, our results could reflect the impact of dysglycemia on TB, thereby limiting the causal inferences that we can draw around directionality.

## CONCLUSION

Young persons with LTBI had higher levels of inflammation but similar cardiometabolic profiles as compared with their peers uninfected with TB. Unlike that in adults, *Mtb* infection in young persons may not be associated with cardiometabolic derangement, although the prognostic relevance of chronically elevated hsCRP remains uncertain. However, larger confirmatory studies are required, especially in sub-Saharan Africa and Southeast Asia, as are detailed immune mechanistic studies with deep cardiometabolic phenotyping. These will be key to better understanding of the relationship between *Mtb* infection and risk of future cardiometabolic syndromes.

## Supplementary Material

ofaf194_Supplementary_Data

## Supplementary Data

Supplementary materials are available at *Open Forum Infectious Diseases* online. Consisting of data provided by the authors to benefit the reader, the posted materials are not copyedited and are the sole responsibility of the authors, so questions or comments should be addressed to the corresponding author.

## Notes

***Author contributions.*** I. M. M.: conceptualization, investigation, data curation, data analysis and visualization, writing–original draft, and writing–review and editing. N. A. B. N., J. J., B. L. C., M. J. S., H. J. Z.: investigation and writing–reviewing and editing. B. L. C.: investigation, writing–reviewing and editing, and data analysis. K. A. W., R. J. W.: conceptualization, investigation, writing–review and editing, and supervision. All authors read and approved the final manuscript.

***Data sharing.*** Data are publicly available at https://wwwn.cdc.gov/nchs/nhanes/default.aspx. Programming code is available upon request.

***Disclaimer.*** For the purposes of open access, the authors have applied a CC-BY public copyright to any author accepted manuscript arising from this submission.

***Financial support.*** No external funding was received for this study. R. J. W. and K. A. W. are funded by the Francis Crick Institute, which is supported by Cancer Research UK (FC2112), Medical Research Council (FC2112), and Wellcome (FC2112). R. J. W. also receives support from Wellcome (226817) and the NIHR Biomedical Research Center of Imperial College NHS Trust. M. J. S. receives support from the US National Institutes of Health (K24 HL166024). H. J. Z. is supported by the South African Medical Research Council and by the National Institutes of Health (R01HD074051). N. A. B. N. gratefully acknowledges support from the South African Medical Research Council, National Research Foundation, the US National Institutes of Health, UK Medical Research Council, and the Lily and Ernst Hausmann Trust.

***Potential conflicts of interest.*** All authors: No reported conflicts.
