# Supplementary material for: Cardiometabolic Biomarkers and Systemic Inflammation in US Adolescents and Young Adults With Latent Tuberculosis Infection: A Population-Based Cohort Study
Source: Open Forum Infect Dis. 2025 Mar 28;12(4):ofaf194. doi: 10.1093/ofid/ofaf194 (PMC12002011; doi:10.1093/ofid/ofaf194)
Supplement: ofaf194_Supplementary_Data [file ofaf194_supplementary_data.docx]

**Supplementary Material**

**Intermediate cardiometabolic outcomes in US adolescents with latent tuberculosis infection: a population-based cohort study.**

**Table of contents Page**

Figure S1 2

Figure S2 3

Table S1 4

Table S2 5

**Figure S1.** Flow chart of participant inclusion in final analytic sample using US NHANES 1999-2000

Completed tuberculin skin testing (TST)

N = 7,386

(TST negative = 6,976; TST positive = 410)

Age <12 and >30 years (n=4,656)

- TST negative = 4,330
- TST positive = 326

**Alt Text**: PRISMA flow chart of participant inclusion in final analytic sample using US NHANES 1999-2000.

Complete analytic dataset

N = 2,217

Tested TB negative

N = 2,142

Tested LTBI positive

N = 75

12 – 30 years old AND valid TST

N = 2,730

(TST negative = 2,644; TST positive = 86)

Excluded* from analysis (N = 513)

Has BCG scar (n=91)

Missing values in covariates

- Country of birth (n=1)
- Food security (n=56)
- Cotinine (n=142)
- Sedentary time (n=33)
- FPG (n=291)
- HbA1c (n=55)
- Current insulin use (n=6)

**Figure S2.** Covariate balance before and after propensity score matching.

**Alt Text**: two (2) graphs illustrating covariate balance before and after propensity-score matching assessed using standard mean differences and Kolmogorov-Smirnov statistics.

**Table S1.** Main and interaction effects of age and latent tuberculosis infection on cardiometabolic indices among propensity score matched adolescents and young adults (12-30 years old), unweighted US NHANES 1999-2000.

| **Biomarker** | **Effect estimates** | | | | | |
| --- | --- | --- | --- | --- | --- | --- |
|  | ***Mtb* status^†^** | | **Age group^††^** | | ***Mtb* status*Age group^†††^** | |
|  | **β (95%CI)** | **P value** | **β (95%CI)** | **p value** | **β (95%CI)** | **p value** |
| Log hsCRP | 0.63 (0.08, 1.19) | 0.026 | 0.68 (0.35, 1.0) | <0.001 | 0.12 (0.01, 0.25) | 0.031 |
| Log NLR | 0.10 (-0.06, 0.27) | 0.23 | 0.27 (0.17, 0.37) | <0.001 | -0.12 (-0.35, 0.10) | 0.29 |
| Log Ferritin | -0.28 (-0.63, 0.07) | 0.12 | 0.37 (0.17, 0.58) | <0.001 | 0.49 (0.03, 0.95) | 0.038 |
| FPG (mmol/L) | -0.17 (-0.41, 0.07) | 0.16 | -0.13 (-0.27, 0.02) | 0.091 | 0.23 (-0.11, 0.57) | 0.19 |
| HbA1c (%) | -0.04 (-0.15, 0.07) | 0.44 | -0.04 (-0.10, 0.03) | 0.25 | 0.03 (-0.12, 0.17) | 0.71 |
| Alb1c (%) | -0.56 (-7.47, 6.28) | 0.87 | 1.54 (-2.58, 5.68) | 0.46 | -1.56 (-10.6, 7.52) | 0.73 |
| Fasting insulin (ρmol/L) | -8.7 (-35.5, 18.0) | 0.52 | -19.4 (-35.8, -2.9) | 0.022 | -1.63 (-39.8, 36.5) | 0.93 |
| Fasting c-peptide (nmol/L) | -0.05 (-0.23, 0.12) | 0.55 | -0.03 (-0.14, 0.08) | 0.62 | -0.01 (-0.26, 0.25) | 0.97 |
| HOMA2-IR | -0.09 (-0.46, 0.28) | 0.64 | -0.01 (-0.59, 0.48) | 0.97 | -0.06 (-0.59, 0.48) | 0.84 |

^†^ *Mtb* uninfected (reference group) versus LTBI.

^††^ 12-15 years old (reference group) versus 16-30 years old.

**^†††^** Mtb status*Age group = interaction term.

LTBI = latent tuberculosis infection; Log = natural logarithm; hsCRP = high sensitivity C-reactive protein; NLR = neutrophil lymphocyte ratio; FPG = fasting plasma glucose; Alb1c = glycated albumin; HbA1c = glycated hemoglobin; HOMA2-IR = homeostatic model assessment of insulin resistance.

**Table S2.** Characteristics of adolescent and young adult participants (12-30 years old) according to latent tuberculosis infection status before and after propensity score matching, US NHANES 2011-2012

| **Characteristic** | **Unmatched** | | | **PS matched** | | |
| --- | --- | --- | --- | --- | --- | --- |
|  | ***M.tb* uninfected** | **LTBI** | **SMD** | ***M.tb* uninfected** | **LTBI** | **SMD** |
| Number | 1,590 | 69 |  | 276 | 69 |  |
| Age (years) | 19.6 (5.5) | 21.5 (5.4) | 0.35 | 21.9 (5.6) | 21.5 (5.4) | 0.068 |
| 12-15 years old | 473 (29.7%) | 12 (17.4%) | 0.29 | 47 (17.0%) | 12 (17.4%) |  |
| 16-30 years old | 1,117 (70.3%) | 57 (82.6%) |  | 229 (83.0%) | 57 (82.6%) | 0.010 |
| Female sex | 781 (49.1%) | 30 (43.5%) | 0.13 | 128 (46.4%) | 30 (43.5%) | 0.058 |
| Race/ethnicity |  |  |  |  |  |  |
| Hispanic | 420 (26.4%) | 24 (34.8%) | 0.18 | 101 (36.6%) | 24 (34.8%) | 0.048 |
| Non-Hispanic | 1,170 (73.6%) | 45 (65.2%) |  | 175 (63.4%) | 45 (65.2%) |  |
| Country of birth |  |  |  |  |  |  |
| USA | 1,311 (82.5%) | 17 (24.6%) | 1.42 | 69 (25.0%) | 17 (24.6%) | 0.008 |
| Non-US | 279 (17.5%) | 52 (75.4%) |  | 207 (75/0%) | 52 (75.4%) |  |
| Household food security |  |  |  |  |  |  |
| Secure | 1,172 (73.7%) | 48 (69.6%) | 0.092 | 203 (73.6%) | 48 (69.6%) | 0.088 |
| Insecure | 418 (26.3%) | 21 (30.4%) |  | 73 (26.4%) | 21 (30.4%) |  |
| Sedentary time (hours/day) | 3.0 (2.0, 4.0) | 3.1 (2.2, 4.3) | 0.007 | 3.0 (1.9, 4.1) | 3.1 (2.2, 4.3) | 0.012 |
| Cotinine (ng/dL) | 0.05 (0.01, 0.98) | 0.06 (0.02, 0.22) | 0.19 | 0.03 (0.01, 0.30) | 0.06 (0.02, 0.22) | <0.001 |
| BMI (kg/m^2^) | 25.6 (6.8) | 25.9 (6.6) | 0.051 | 25.7 (7.3) | 25.9 (6.6) | 0.032 |

Values are mean (SD) or number (%) or proportion (%) or median (25^th^, 75^th^ percentile).

PS = propensity score; SMD = standardized mean difference; BMI = body mass index.

Matched on age, sex, race, country of birth, household food, LTPA, serum cotinine, and BMI.
